# Avian Influenza Knowledge among Medical Students, Iran

**DOI:** 10.3201/eid1404.070296

**Published:** 2008-04

**Authors:** Kamyar Ghabili, Mohammadali M. Shoja, Pooya Kamran

**Affiliations:** * Tabriz University of Medical Sciences, Tabriz, Iran

## Abstract

Avian Influenza Knowledge among Medical Students, Iran

**To the Editor:** Avian influenza is an infectious disease caused by type A strains of influenza virus (*1*). Since January 2004, Thailand and several other Southeast Asian countries have experienced outbreaks of avian influenza in poultry, and >100 million poultry have been culled or have died (www.who.int/csr/disease/avian_influenza/en). The prevalence of severe and fatal cases involving bird-to-human transmission is increasing (*2*). Experts fear that the avian influenza virus now circulating in Asia will mutate into a highly infectious strain and pass not only from animals to humans, but also among humans, which would lead to a pandemic (*3*).

During a pandemic, public health agencies and medical students will play critical roles in controlling the spread of disease (*4*). Therefore, medical school curricula should include specific courses in the epidemiology of avian influenza to ensure that all medical students and health care professionals will have the knowledge needed to confront a potential pandemic. In Iran, medical education comprises basic sciences (first to third year), externship (fourth to fifth year, preclinical education), and internship (sixth to seventh year). Medical students study virology during the second year of medical school. Thereafter, no additional coursework in virology is offered. Because several cases of avian influenza have been found in adjacent countries such as Turkey and Iraq, we anticipate that the virus will spread to Iran. Therefore, we designed a study to assess the knowledge of a group of Iranian medical students regarding avian influenza and to delineate the potential source of their knowledge.

The study population comprised second- and third-year medical students at the Faculty of Medicine, Tabriz University of Medical Sciences, in May 2006. We used a self-administered questionnaire that was based on information obtained from a review of the literature on avian influenza. This questionnaire (Table) comprised 3 sections: 1) demographic information, including age and sex of participants (2 items); 2) avian influenza–related questions covering general information, history, modes of transmission, clinical symptoms, and prevention (18 items); and 3) a multiple-choice question regarding the students’ source of information about avian influenza (1 item). (As shown, the questionnaire used the common parlance “bird flu” for avian influenza.) Possible responses for section 2 included “yes,” “no,” and “I don’t know.” The knowledge score was calculated by giving +1 for a correct answer, –1 for an incorrect answer, and zero for “I don’t know” responses. A total of 18 points could be achieved if all questions in section 2 were correctly answered. Higher scores indicated a greater level of knowledge. We invited 2 epidemiologists and 1 statistician to qualify and examine the questions. Data were presented in mean ± standard deviation or percentage when appropriate. Statistical analysis was performed by SPSS Windows version 12.0 (SPSS Inc., Chicago, IL, USA) using the χ^2^ test; p value was set at 0.05.

**Table:** Respondents’ knowledge of avian influenza (n = 234), Iran, May 2006*

Questions	Correct answer	% Yes	% No	% Don’t know
History				
1. The first case of human infection with bird flu virus occurred in Hong Kong in 1997.	Yes	27.7	3.2	69.1
2. Most fatal cases of bird flu have been reported in Vietnam.	Yes	28	2.2	69.9
General information				
3. Influenza virus occurs naturally among wild birds.	Yes	14.3	63.7	22
4. Bird flu may be transmitted into other mammals such as horses and pigs.	Yes	25.3	19.8	54.9
Transmission				
5. Transmission of the disease from person to person is possible.	Yes	47.3	19.8	33
6. Main modes of transmission are through saliva and nasal secretions.	Yes	54.2	13.5	32.3
7. Bird flu virus can be transmitted into persons through the alimentary tract.	No	74.2	15.7	10.1
8. Bird flu is transmitted into humans through handling and cleaning of contaminated game.	Yes	41.3	37	21.7
9. The consumption of contaminated chicken as broiler would have the risk of affliction.	Yes	72.2	21.1	6.7
10. Cooking eggs as soft-boiled eliminates the virus.	No	19.1	68.5	12.4
Diagnosis				
11. A laboratory test is needed to confirm bird flu in humans.	Yes	10	83	7
Clinical presentations				
12. Respiratory tract is the main infected system in the body.	Yes	59.8	9.2	31
13. The incubation period of bird flu is ≈7 days.	Yes	13.6	2.3	84.1
14. Symptoms of bird flu in humans are similar to seasonal influenza.	Yes	20	11	69
15. Bleeding from the nose and bleeding from the gums are early symptoms of bird flu.	Yes	2	13	85
16. Bloody diarrhea (dysentery) is one of the manifestations of bird flu.	No	30.8	8.8	60.4
Prevention				
17. Bird flu is a preventable infection.	Yes	86	4.3	9.7
18. There is a vaccine to protect humans from bird flu virus.	No	20.9	37.4	41.8

Two hundred thirty-four of 252 second- and third-year medical students completed the questionnaire. The mean age of the respondents was 19 ± 0.87 years (range 18–23). Twenty-nine percent (n = 68) of the students were male and 71% (n = 166) were female.

The mean knowledge score was 4.76 of 18 (total of correct and incorrect responses) (range –6 to 11). Second- and third-year students comparably responded to 16/18 questions (χ^2^ test). A list of questions and the percentage of students’ responses are provided in the Table.

Most of the respondents (67.2%) indicated that mass media (radio, television, and newspapers) was their major source of information about avian influenza, followed by scientific books and journals (8.3%), the Internet, (13%), and family and friends (10.4%). Only 1.1% of the medical students mentioned “school educational materials” as the source of their information.

Our study shows a relatively low level of knowledge of avian influenza among a group of Iranian medical students. Surprisingly, mass media was the main source of information in our study. Training health care professionals as well as medical students is of great importance in controlling infectious diseases. The findings of this study should be considered seriously by local health centers and disease control agencies because preparing health care professionals with sufficient knowledge is essential to confronting a potential pandemic. We believe that the low level of knowledge about avian influenza among these medical students is primarily a reflection of insufficient academic courses in the medical school curriculum. We strongly recommend improving the quality of education on this topic through access to textbooks, articles, seminars, and specific courses.
